# Evaluating Autologous Platelet Aggregate as a Scaffold in the Treatment of Human Permanent Molars With Pulpitis: A Case Series

**DOI:** 10.7759/cureus.69004

**Published:** 2024-09-09

**Authors:** Alpa Gupta, Dax Abraham, Vivek Aggarwal, Shakila Mahesh

**Affiliations:** 1 Conservative Dentistry and Endodontics, Manav Rachna International Institute of Research and Studies, Faridabad, IND; 2 Conservative Dentistry and Endodontics, Manav Rachna Dental College and Hospital, Faridabad, IND; 3 Conservative Dentistry and Endodontics, Faculty of Dentistry, Jamia Millia Islamia, New Delhi, IND; 4 Microbiology, Manav Rachna Dental College and Hospital, Faridabad, IND

**Keywords:** concentrated growth factors, growth factors, pulpitis, pulpotomy, reversible pulpitis

## Abstract

This case series aims to investigate the radiographic and clinical results of pulpotomy using concentrated growth factor (CGF), a scaffold obtained from the host, on adult human permanent molars. A total of four cases diagnosed with symptomatic reversible pulpitis based on history, clinical examination, and investigation were planned for CGF pulpotomy. Blood required for the procedure was collected in a 10 ml test tube without anticoagulants to produce CGF. Subsequently, the sample was promptly centrifuged using a tabletop centrifuge. The clot-free pulp chamber was then shielded with a small CGF membrane fragment. A layer of mineral trioxide aggregate (MTA), approximately 2 mm thick, was applied over the CGF membrane. Following this, the tooth was temporized with glass ionomer cement. The patients were scheduled for a follow-up visit after a day to assess postoperative discomfort and to proceed with the final composite restoration. All the patients were recalled at 6 and 12 months for follow-up. Three of the patients were clinically and radiographically asymptomatic following the treatment. The tooth demonstrated a normal periodontal ligament space on radiographic inspection, and it passed pulp sensibility tests. One patient, though, complained of excruciating discomfort 24 hours after the procedure. The favorable results of the three instances imply that more investigation is required to validate the application of this biocompatible alternative to the management of pulpitis in permanent human molar teeth. More research with larger sample numbers and longer recollection periods is required. The use of concentrated growth factor (CGF), derived from the patient's own blood, serves as an excellent biological scaffold for the treatment of pulpal diseases.

## Introduction

Maintaining dental health and the vitality of the dentin-pulp complex are essential to therapeutic treatment approaches. One type of tooth damage that entails intricate interactions between the tooth's repair, regeneration, and damage mechanisms is dental caries. Though it is usual to consider each of these processes separately, the vitality of the tissue and the lifetime of the tooth ultimately depend on how these processes combine and are balanced [1].

The pulp must be shielded when repairing carious exposures in immature permanent teeth or the intricate root canal systems of primary molars [2]. Exposures might result from dental caries, iatrogenic errors, or acute traumas [3]. Pulpotomy is a widespread treatment for primary teeth [2] and entails the surgical removal of a section of the coronal pulp tissue and the application of a substance to the remaining radicular tissue that promotes and accelerates recovery while also shielding the pulp from further harm.

Various materials have been suggested to promote the production of dentine bridges through the dentinogenic potential of pulpal cells [4]. Stanley (1989) advocated for the use of calcium hydroxide as an essential pulp therapy, and this material is being used today to protect exposed dental pulps [5].

Pulp exposures to pulpitis can heal from leftover radicular tissue and pulpotomy agents, especially mineral trioxide aggregate (MTA), are particularly biocompatible; therefore, several case studies have suggested pulpotomy as a potential treatment [6]. As a result, developing biocompatible treatments to maintain pulp vitality and prolong tooth life is essential. Novel biologically based therapies that reduce pulp inflammation and increase dentine pulp tissue development are urgently needed to increase the success rate.

The first report of platelet-rich fibrin (PRF) produced from the host was recorded in a paper by Choukroun et al. (2006). It is referred to as a second-generation platelet concentrate and is superior to conventional platelet-rich plasma (PRP) in several ways. The principal advantages of this preparation are its ease of use and 100% autologous nature due to the lack of biochemical blood processing. Several case reports have considered the use of PRF as a pulpotomy agent [7].

Concentrated growth factor (CGF) is the most recent generation of platelet concentrate products, and it is likewise a host-derived product [8]. Contrary to PRP, CGF is devoid of anticoagulants and bovine thrombin, which could result in immunological rejection and cross-infection [9]. It was made from PRF by speeding up the centrifugation process, which converted fibrinogen into fibrin. This may strengthen the matrix's tensile strength and promote the release of growth factors and platelets [9-11].

In terms of composition and processing, CGF performs better than PRP and PRF as a biomaterial [9, 10]. Because of its autogenesis, clinical use, and biodegradability, CGF has gained favor in the fields of periodontal regeneration, facial reconstruction, and dental implants for mending defects in the bone and promoting soft-tissue healing [12].

CGF also possesses stronger adhesive strength, tensile strength, and viscosity, which aid in the regeneration process, as well as immune cells that are beneficial in managing inflammation and reducing the danger of infection [13]. This case series highlights the use of a host-derived scaffold with CGF as a pulpotomy agent in four permanent human molar teeth diagnosed with symptomatic reversible pulpitis.

## Case presentation

Case 1

**Figure 1:**
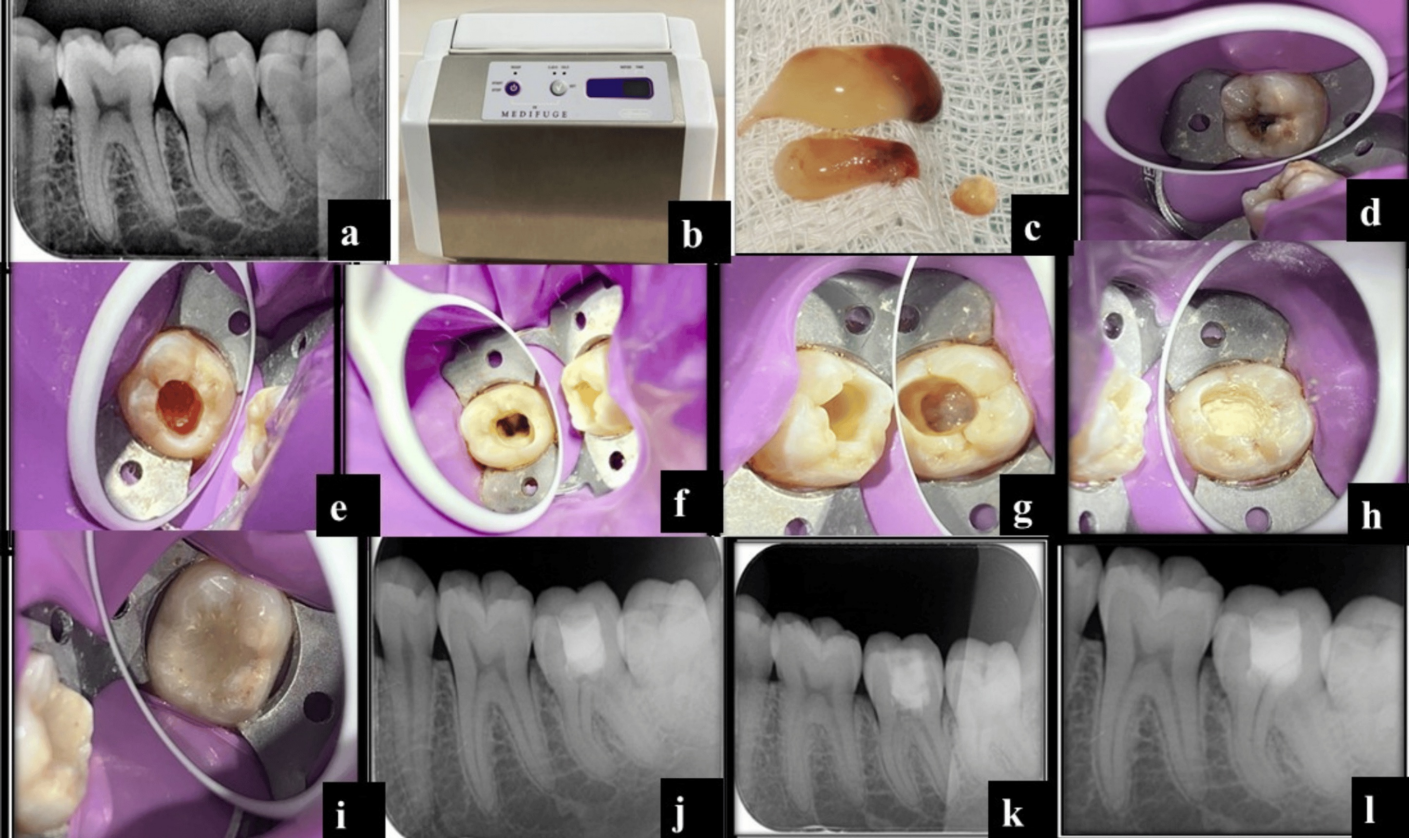
Preoperative radiograph (a); CGF machine (b); prepared CGF (c); clinical picture of the affected tooth (d); removal of coronal pulp (e); hemostasis achieved (f); placement of CGF membrane (g); placement of MTA (h); final restoration (i); postoperative radiograph (j); 6-month follow-up radiograph (k); 12-month follow-up radiograph (l) MTA: mineral trioxide aggregate

A 23-year-old female patient reported to our institution with a chief complaint of pain in the lower back tooth region for two to three days. On clinical examination, occlusal caries were seen on the left mandibular second molar. An intra-oral periapical radiograph revealed radiolucency involving enamel, more than one-third of dentin, and approaching pulp. Periapically, no radiolucency was seen with the mesial and distal roots (Figure 1a). Pulp sensibility testing of the involved tooth with electronic pulp stimulation and cold test yielded a positive response. From the clinical assessment and radiographic findings, a diagnosis of symptomatic reversible pulpitis was made and coronal pulpotomy using CGF to treat tooth #37 was suggested to the patient as an alternative treatment to root canal therapy. Following the patient's written consent, the necessary blood was drawn into a 10 ml test tube without the use of an anticoagulant to prepare CGF, which was then quickly centrifuged using a tabletop centrifuge (MEDIFUGE CGF, Silfradent, Santa Sofia, Italy) (Figure 1b). The machine's "preprogramming" allowed for the automatic adjustment of each of these procedures. The final product was composed of three layers: red blood corpuscles at the bottom of the tube, a fibrin clot in the middle, and acellular platelet-poor plasma at the top.

Using a sterile syringe, the topmost layer which was devoid of platelets was extracted from the three layers that had formed. The CGF-containing fibrin gel layer was isolated from the layer of red blood cells. A hemostatic clamp was used to hold the concentrated growth membrane-containing layer in place while microsurgical scissors were used to detach it from the red blood cell layer. After that, the CGF layer was compressed to create a CGF membrane (Figure 1c). The same protocol is followed in all the cases for the preparation of CGF.

Tooth #37 was isolated with a rubber dam and anesthetized with 2% lidocaine and 1/80,000 adrenaline (Astra Zeneca Pharma India Ltd., Bangalore, India). Using a round bur and a high-speed handpiece with abundant irrigation, a pulpotomy was carried out, removing coronal pulp tissue all the way to the pulp chamber floor. Irrigating the cavity with cotton pellets and sterile saline allowed for hemostasis to be accomplished (Figures 1d-1f). A tiny piece of CGF membrane was applied to the pulpal wound free of blood clots (Figure 1g). A layer of MTA about 2 mm thick was applied over the CGF membrane (ProRoot, Dentsply Tulsa Dental Specialty, Tulsa, OK), followed by GIC after the initial setting of MTA (Figure 1h). After a day, the patient was recalled for final composite restoration and to assess any postoperative pain (Figures 1i-1j). As follow-ups, radiographs at 6, 12, and 24 months were taken after the pulpotomy procedure. The patient reported no pain or discomfort, and the tooth responded satisfactorily to pulp tests 6 and 12 months following recall. The radiographic examination (Figures 1k-1l) revealed a normal periodontal ligament space and trabecular bone pattern.

Case 2

**Figure 2:**
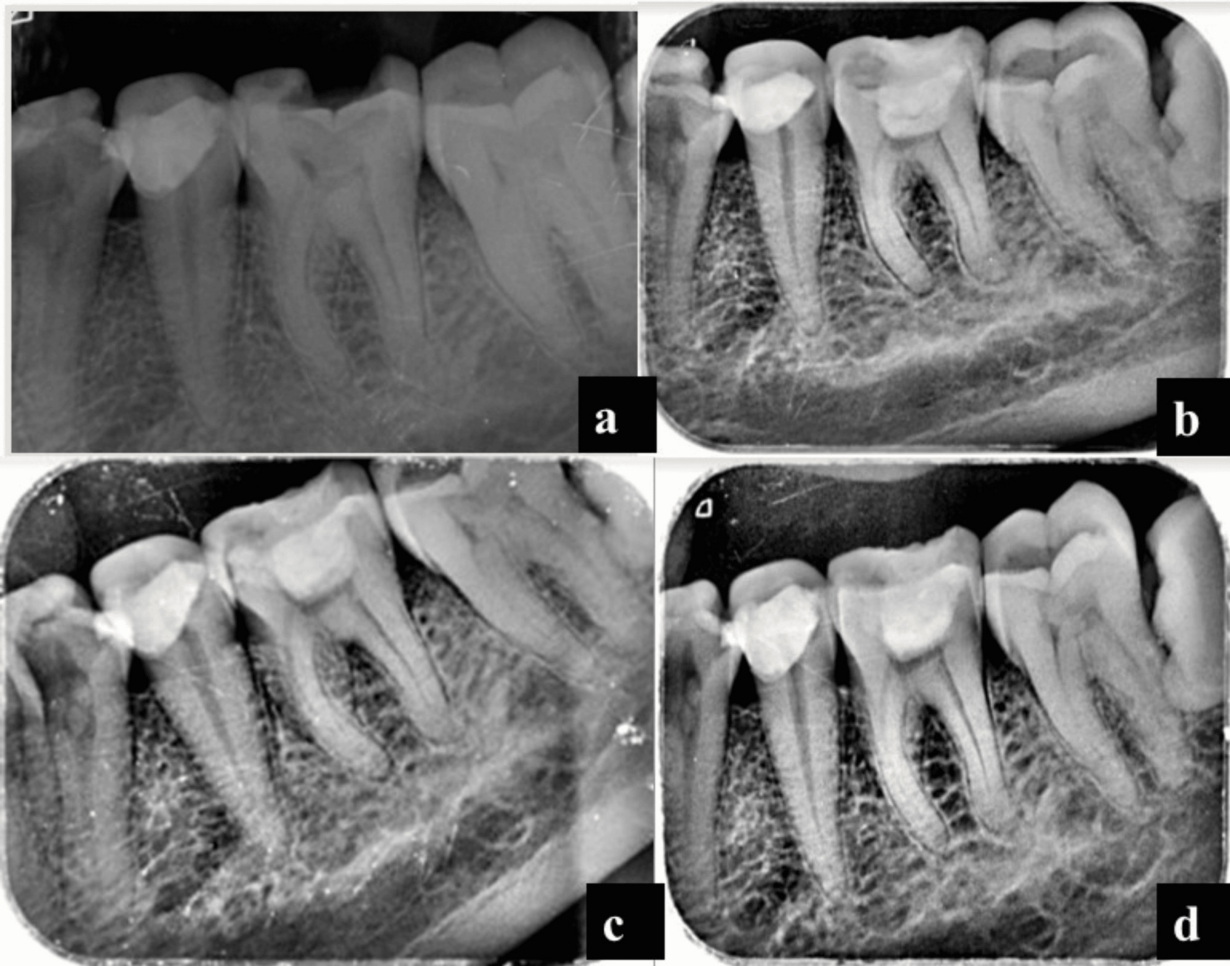
Preoperative radiograph (a); postoperative radiograph (b); 6-month follow-up radiograph (c); 12-month follow-up radiograph (d)

Herein, a 35-year-old female patient reported pain in the left lower back tooth region for a week prior to presentation. The pain was aggravated only by cold stimuli and was non-lingering. On clinical examination, there was a deep caries lesion on tooth #36 and a diagnosis of symptomatic reversible pulpitis was made. After an explanation of the treatment plan, written informed consent was obtained. CGF was prepared from the blood sample as per standard protocol. Local anesthesia was administered and rubber dam isolation was achieved. A sterile diamond point was used to excavate the caries and coronal pulpotomy was performed, then a small piece of CGF membrane was placed over the pulpal exposure site, which was covered with 2 mm of MTA. The teeth were temporized. The patient was recalled after three days for re-evaluation and final composite restorations (Figures 2a-2b). The patient was asymptomatic at the 6- and 12-month follow-ups (Figures 2c-2d).

Case 3

**Figure 3:**
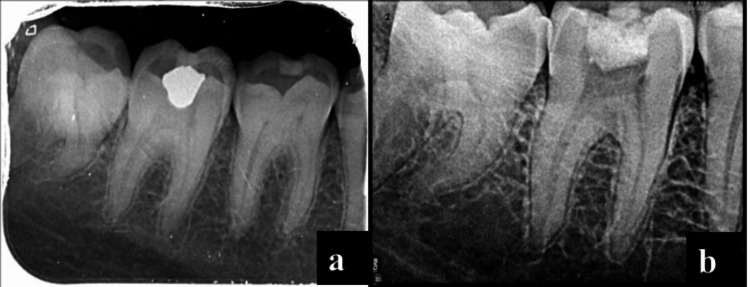
Preoperative radiograph (a); access opening radiograph (b)

A 30-year-old male patient reported a broken filling in the right lower back tooth region for 15 days, which was associated with pain from hot/cold sensations, and experienced relief as soon as the stimuli were removed. There was dislodged restoration and secondary caries in tooth #47, which showed a positive response to pulp sensibility tests (both cold test and EPT). Hence this was diagnosed as symptomatic reversible pulpitis. After an explanation of the treatment plan, written informed consent was obtained. CGF was prepared from the blood sample as per standard protocol. After local anesthesia and rubber dam isolation caries were excavated, and pulpal exposure was seen. Coronal pulpotomy was performed, a small piece of CGF membrane and 2 mm of MTA were placed, and the tooth was restored with temporary filling material (Figures 3a-3b). However, the patient was symptomatic within 24 hours with complaints of severe pain and tenderness despite taking analgesics. It was considered that the pulpotomy had failed, and the root canal procedure was started on the next day. Access opening was performed and pulp was extirpated under local anesthesia. Complete chemo-mechanical preparation was performed and standard disinfection protocol was followed using 3% hypochlorite. Obturation and post-endodontic restoration with composite were performed after three days, once the tooth became asymptomatic.

Case 4

**Figure 4:**
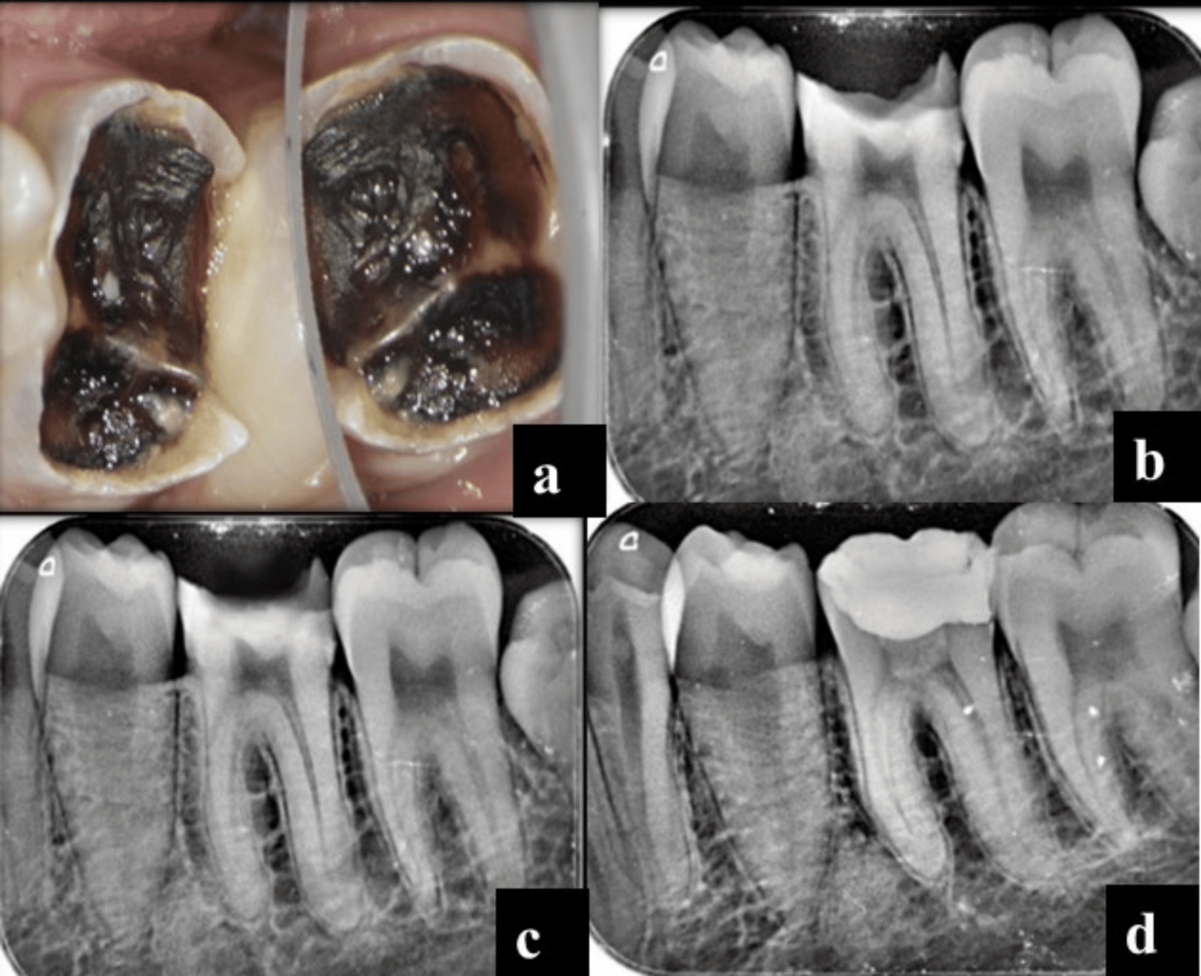
Preoperative clinical picture (a); preoperative radiograph (b); postoperative radiograph (c); 12-month follow-up (d)

A 24-year-old woman presented with a pain-free, carious tooth in the left lower back tooth region. On examination, occlusal caries was present in tooth #36, pulp sensibility tests were not significant, and no periapical lesion was identified radiographically. After an explanation of the treatment plan, written informed consent was obtained. CGF was prepared from the blood sample as per standard protocol. After rubber dam isolation, caries excavation was done, leading to pulpal exposure. Pulpal bleeding was controlled with a cotton pellet dampened with 3% hypochlorite. CGF membrane was placed, followed by two mm of MTA placement; after this, a temporary restoration was placed. The final composite restoration was placed after three days (Figures 4a-4c). Clinically and radiographically, the patient remained asymptomatic during the 12-month follow-up assessments (Figure 4d).

## Discussion

A common treatment for teeth with partially formed roots that reveal the pulp is called a pulpotomy. It has been suggested that in permanent teeth, pulpal tissue removal and root canal therapy can be difficult and time-consuming for both the patient and the physician and it is not always economical. Moreover, the outcome of a subsequent root canal procedure for the tooth remains unaffected by the failure of crucial pulp therapy [14-15]. However, more investigation is required to evaluate pulpotomy treatment for permanent adult teeth. 

Recently, MTA was used for pulpotomy in primary molars [15], and it has demonstrated excellent sealing ability, robust biocompatibility, and stimulation of pulpal tissue repair [16]. Several laboratory studies have been carried out to assess the biocompatibility of MTA by analyzing several parameters like proliferation and viability, utilizing diverse cell types in direct or indirect contact with MTA. Because of its high pH, MTA shows increased cytotoxicity in its early mixing condition [17-18]. Thus, it is crucial to discover biocompatible treatments to preserve pulp vitality and extend tooth life [19].

One such treatment based on biologically generated products from the host is PRF. Second-generation platelet concentrations, such as PRF, are frequently used to expedite the repair of both soft and hard tissues. Its advantages over PRP are its low cost, lack of biochemical change, and ease of preparation and application. CGF differs from PRF regarding the amount of growth factor release, which is more with CGF.

In the present case series, an effort was made to use these growth factors to promote the repair of teeth with pulpitis. As previously mentioned, CGF was created with the patient's own blood and placed in the pulp chamber after a pulpotomy procedure. A coating of MTA was applied. Over the MTA, the glass-ionomer cement was placed, and it was eventually replaced with a composite restoration. Because MTA is hydrophilic and needs moisture to set, it was selected because these properties are useful in clinical situations where there may be a risk of moisture contamination [20]. Additionally, a second coronal seal was created to get rid of microleakage. At six and 12 months, the tooth showed no symptoms and responded well to sensitivity testing in three of the cases. Subsequent radiographs showed a nearly normal trabecular pattern and total elimination of the periapical rarefactions. However, Case 3 failed immediately after CGF pulpotomy. This could be attributed to the extent of pulpal inflammation. For the pulpotomy procedure to be successful, it is imperative for the pulp to be in the reversible stage. Currently, the diagnosis of reversible and irreversible pulpitis is based on history, radiographic findings, and clinical examinations including sensibility tests which might not give the accurate status of pulp in every case. However, CGF is a biologically safe alternative for pulpotomy in permanent molars.

## Conclusions

Within the observations of these clinical cases, it can be concluded that CGF can be a safe and effective approach for pulp therapy procedures. Autologous platelet aggregates can be considered as an alternative to synthetic scaffolds for pulp therapies. CGF, a newer host-derived scaffold, is highly beneficial in pulpotomy procedures. Given the benefits of CGF, further research ought to be done to demonstrate its superior clinical efficacy over competing treatments. Clinical trials with a large sample size are required to prove its superior clinical efficacy.
